# Integrated CO_2_ capture and reverse water–gas shift reaction over CeO_2_-CaO dual functional materials

**DOI:** 10.1098/rsos.230067

**Published:** 2023-04-05

**Authors:** Shuzhuang Sun, Chen Zhang, Sining Chen, Xiaotong Zhao, Yuanyuan Wang, Shaojun Xu, Chunfei Wu

**Affiliations:** ^1^ School of Chemistry and Chemical Engineering, Queen's University Belfast, Belfast BT7 1NN, UK; ^2^ Department of Chemistry, University College London, 20 Gordon Street, London WC1H 0AJ, UK; ^3^ UK Catalysis Hub, Research Complex at Harwell, Didcot OX11 0FA, UK; ^4^ Department of Chemical Engineering, University of Manchester, Manchester M13 9PL, UK; ^5^ Cardiff Catalysis Institute, School of Chemistry, Cardiff University, Cardiff CF10 3AT, UK

**Keywords:** integrated CO_2_ capture and utilization, reverse water–gas shift reaction, dual functional materials, carbon capture, CaO

## Abstract

Achieving carbon neutrality is one of the most important tasks to meet the environmental challenges due to excessive CO_2_ emissions. Integrated CO_2_ capture and utilization (ICCU) represents an effective process for direct utilization of CO_2_-contained exhaust gas (e.g. flue gas), in which converting the captured CO_2_ into CO via reverse water–gas shift (RWGS) reaction is a promising route. The dual functional materials (DFMs), containing CO_2_ adsorbents and catalysts, are widely applied to achieve ICCU. The conventional active metals (Ni, Fe, etc.)-based DFMs and non-transition metal DFMs (e.g. CaO) are restricted by low CO selectivity, catalytic efficiency or CO generation in the CO_2_ capture step. To address the above obstructs in the application of DFMs, the metal oxides-based DFMs, MO_x_-CaO (M = Al, Ce, Ti or Zr), are synthesized and evaluated. The CeO_2_-CaO outperformed the other metal oxides-based DFMs and possessed significantly improved catalytic performance. It is found that 33% CeO_2_-CaO DFM displayed approximately 49% CO_2_ conversion and approximately 100% CO selectivity in integrated CO_2_ capture and reverse water–gas shift reaction (ICCU-RWGS) at 650°C, while CaO-alone only achieved approximately 20% CO_2_ conversion at the same condition. The surface basicity of CeO_2_ is revealed to contribute to the improved catalytic performance by enhancing CO_2_ chemisorption and activation in the hydrogenation step. Furthermore, CeO_2_-CaO material possessed excellent cycle stability in 20 cycles ICCU-RWGS, achieving a sustainable and high-efficient performance in CO_2_ conversion and CO selectivity.

## 1. Introduction

Numerous countries have pledged to achieve carbon neutrality around the mid-twenty-first century to eliminate the severe greenhouse effect and accompanying environmental issues [1]. However, in the foreseeable future, it is still inevitable to use fossil fuels to meet the energy demands [2], which would emit a huge amount of CO_2_. Carbon dioxide capture and utilization or storage (CCUS) processes are believed to be effective and essential solutions to meet the great challenges for carbon neutrality [3–6]. However, the high capital costs of CCUS processes [7], including CO_2_ enrichments, transportation and heat management, obstruct the industrial deployments.

To directly use the diluted CO_2_ in the exhaust gas, integrating CO_2_ capture with utilization (ICCU) [8–10] into one process exhibits an impressive performance and attractive application potential [11]. Specifically, the ICCU process can be achieved by swinging the inlet gas between exhaust gas (e.g. flue gas) and reducing agent (e.g. H_2_) isothermally over the dual functional materials (DFMs). The majority of researchers paid attention to converting captured CO_2_ into CH_4_ [8,12–16], which is mainly re-used as fuel with equivalent carbon emissions. Recently, researchers have achieved CO generation via ICCU by reverse water–gas shift reaction (ICCU-RWGS) [17–20]. The unconsumed H_2_ mixed with CO (syn-gas) can be further introduced into the Fischer–Tropsch synthesis process to produce high-end chemicals (e.g. olefins), which possess a longer life cycle compared with fuels [21]. Various metal-functionalized CaO DFMs were demonstrated to realize ICCU-RWGS, such as Ni-CaO [18,19,22,23], Fe_x_Co_y_Mg_10_CaO [17], FeCrCu/K/hydrotalcite [24] and Fe-CaO [19,25]. Although the introduction of metals (e.g. Ni, Fe or Co) contributes to the effective catalytic RWGS in ICCU, the CO selectivity and undesirable CO generation during CO_2_ capture restrict the further deployment [19]. Specifically, the CO generation in the CO_2_ capture process is attributed to the reaction between CO_2_ and reduced metallic metal (e.g. Fe) [25]. In recent study, removing transition metals from DFMs shows reduced CO generation during CO_2_ capture and improved CO selectivity during the conversion of adsorbed CO_2_ [26,27]. However, the absence of active metals significantly reduced the hydrogenation efficiency of DFMs and further restricted the cycle efficiency of ICCU. In short, there is a trade-off between impurity (i.e. CO) generation in the CO_2_ capture process and the catalytic efficiency and CO selectivity in the RWGS process. It is necessary to develop novel DFMs to avoid CO generation in the CO_2_ capture process while achieving enhanced RWGS efficiency with excellent CO selectivity.

**Figure 1.**
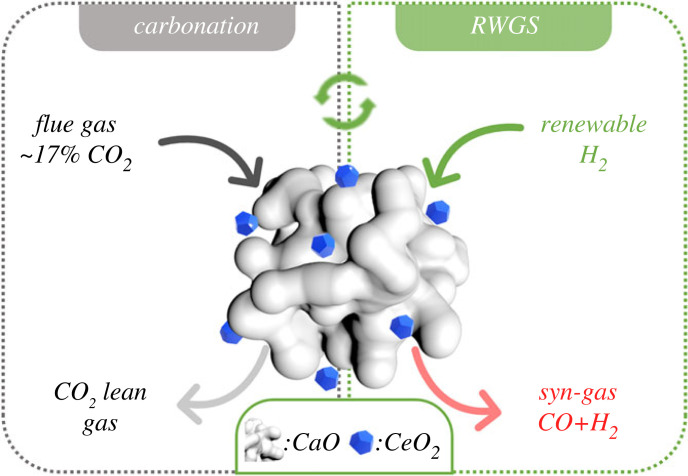
Schematic diagram of ICCU-RWGS.

Metal oxides (such as CeO_2_ and TiO_2_) are believed to be catalytically active in many reaction processes [28], such as reforming processes, photocatalysis and water–gas shift reaction [29]. Those metal oxides so far are mainly applied in ICCU by acting as the catalyst support for the active metal species [18,30,31]. However, there is still a knowledge gap in understanding the catalytic roles and other promotion effects of the metal oxides in DFMs during the ICCU process. Herein, we investigated ICCU performance over the DFMs composing various metal oxides (CeO_2_, TiO_2_, ZrO_2_ and Al_2_O_3_)-CaO DFMs, in which CeO_2_ is identified as the active metal oxide, while other metal oxides are benchmarks. The DFMs were produced by physically mixing the metal oxides and CaO. As illustrated in figure 1, the DFMs firstly act as the adsorbents to reduce the CO_2_ emissions via carbonation, subsequently, the carbonated DFMs are converted in the H_2_ atmosphere with the formation of CO. The catalytic performances of various DFMs and cycle stability were real-time studied using an online gas analyser and discussed with characterizations, in order to reveal the effect of the non-active metal containing CeO_2_-CaO DFM on promoting the ICCU-RWGS.

## 2. Experimental section

### 2.1. Preparation of MO_x_-CaO (M = Al, Ce, Ti or Zr) dual functional materials

The CeO_2_ was synthesized using a hydrothermal method as previously reported [15,16]. Specifically, 5.21 g Ce(NO_3_)_3_·6H_2_O (Sigma-Aldrich, 99%) was dissolved in deionized water (30 ml) to prepare a Ce source solution, followed by the dissolution of 57.6 g NaOH (Sigma-Aldrich, 99%) in deionized water (210 ml) to prepare the precipitant. The Ce source was mixed with the precipitant dropwise for 30 min at room temperature to obtain a slurry. The slurry was transferred into a stainless-steel autoclave and kept at 100°C for 24 h. The precipitate was washed and separated by vacuum filtration using distilled water and ethanol to neutrality and dried at 120°C overnight, to produce a yellow powder, labelled as CeO_2_. The ZrO_2_ (Sigma-Aldrich, 99%), TiO_2_ (Sigma-Aldrich, 99.5%), Al_2_O_3_ (Sigma-Aldrich, 99.5%) and CeO_2_ were calcined at 800°C for 2 h with a heating rate of 5°C min^−1^ before mixing with CaO.

The CaO was derived by a sol–gel method as reported in previous literatures [19,25]. Briefly, 23.6 g Ca(NO_3_)_2_·4H_2_O (Sigma-Aldrich, 99%) and 19.2 g citric acid monohydrate (Sigma-Aldrich, 99.5%) were dissolved into 72 ml distilled water, stirred at room temperature at 80°C and dried at 120°C overnight. The sample was ground and calcined at 850°C for 5 h at a heating rate of 5°C min^−1^ to obtain CaO.

The MO_x_ (M = Al, Ce, Ti or Zr) and CaO are physically mixed by grinding (mass ratio: MO_X_ : CaO = 1 : 2) to prepare the MO_x_-CaO DFMs.

### 2.2. Characterizations

X-ray diffraction (XRD) patterns of MO_x_ (M = Al, Ce, Ti or Zr) were measured using a PANalytical Empyrean Series 2 diffractometer with a Cu Ka X-ray source. The CO_2_ temperature-programmed desorption (CO_2_-TPD) patterns of MO_x_ (M = Al, Ce, Ti or Zr) were measured by a Micromeritics Autochem II 2920 analyser equipped with a TCD detector. Briefly, the MO_x_ were *in situ* reduced at 550°C in H_2_ for 1 h and then cooled down to 30°C under He. After adsorbing CO_2_ in a 10% CO_2_/He gas mixture at 30°C, the temperature was increased to 800°C in He at a heating rate of 10°C min^−1^. Scanning electron microscopy coupled with an energy-dispersive X-ray spectrometer (SEM-EDX, FEI Quanta FEG) was used to characterize the morphology and element dispersion.

### 2.3. Integrated CO_2_ capture and reverse water–gas shift reaction evaluation

The ICCU-RWGS performances of MO_x_-CaO DFMs were evaluated on a tubular fixed-bed reactor (stainless-steel tube; 500.0 mm in length and 10.2 mm in inner diameter). The reactor was placed in the middle of the furnace (Elite TSH-2416CG), filled with 0.3 g DFMs catalyst with quartz wool on both end of the catalysts. The thermocouple was placed in the middle of DFMs to control the temperature. The flow rates of the inlet gases were controlled by mass flow meters (OMEGA FMA2300), and the outlet gas (CO_2_, CO and CH_4_) was monitored by an online gas analyser (Kane Autoplus 5).

The typical ICCU-RWGS reaction procedure includes mainly two steps, i.e. carbonation and hydrogenation. In this work, all the DFMs catalysts were pretreated in 100 ml min^–1^ 5% H_2_/N_2_ at 550°C for 1 h to clean the surface of the catalysts and then equilibrated to the defined evaluation temperature in the range of 600–750°C in 100 ml min^–1^ N_2_. In the carbonation step, 100 ml min^–1^ 17% CO_2_/N_2_ (no added steam and O_2_) was introduced for 1700 s to ideally simulate flue gas CO_2_ capture. Subsequently, the hydrogenation step is to switch the gas to 5% H_2_/N_2_ at 100 ml min^–1^ to convert CO_2_ and regenerate the adsorbent. The cycle evaluations were carried out with extra 5 min N_2_ purge among each ICCU-RWGS procedure.

The real-time CO_2_ conversion, CO generation rate and CO selectivity in RWGS were calculated as equations (2.1)–(2.3).

2.1 $$C_{\mathrm{CO}2}=\frac{\mathrm{CO}\;+{\mathrm{CH}}_{4}}{\mathrm{CO}\;+{\mathrm{CH}}_{4}+{\mathrm{CO}}_{2}}\text{\%},$$

2.2 $$Y_{\mathrm{CO}}=\frac{\mathrm{CO}(\text{\%})\times 1.667\;\mathrm{ml}\;s^{-1}}{0.0224\;\mathrm{ml}\;\mu {\mathrm{mol}}^{-1}\times 0.30\;g}$$

2.3 $$\mathrm{and}\;\;S_{\mathrm{CO}}=\frac{\mathrm{CO}}{\mathrm{CO}\;+{\mathrm{CH}}_{4}}\text{\%}\;,$$

where C_CO2_, Y_CO_ and S_CO_ represent CO_2_ conversion (%), CO generation rate (μmol g_DFM_ s^−1^) and CO selectivity (%). The catalytic performance (CO_2_ conversion, CO yield and CO selectivity) throughout the hydrogenation step was evaluated by the integration of real-time CO_2_ conversion, CO generation rate and CO selectivity.

## 3. Results and discussions

### 3.1. Characterizations of MO_x_ (M = Al, Ce, Ti or Zr)

**Figure 2.**
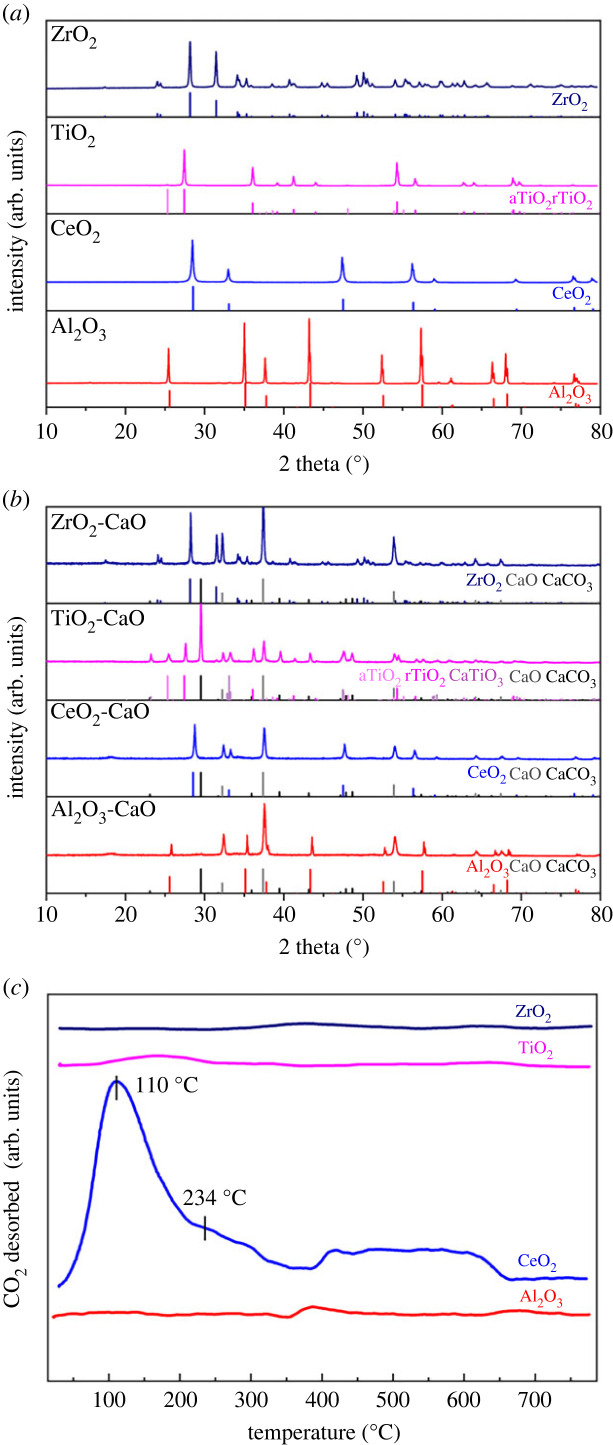
XRD patterns of (*a*) M O_x_ and (*b*) spent M O_x_-CaO; (*c*) CO_2_-TPD profiles of MO_x_ (M = Al, Ce, Ti or Zr).

The XRD patterns of the produced metal oxides (MO_x_) are shown in figure 2*a*. The CeO_2_ and the benchmarks Al_2_O_3_, TiO_2_ and ZrO_2_ all possess pure crystal phase after elevated temperature pretreatment and are consistent with PDF75-1864, PDF78-0694, PDF75-1753 (rutile, rTiO_2_), PDF83-2243 (anatase, aTiO_2_) and PDF86-1451, respectively. Al_2_O_3_, ZrO_2_ and CeO_2_ performed no interaction with CaO during ICCU evaluation, while the TiO_2_ formed a small amount of CaTiO_3_ with CaO (figure 2*b*). The CO_2_-TPD profiles are shown in figure 2*c* to evaluate the basicity of MO_x_. Notably, only CeO_2_ possessed distinct CO_2_ desorption peaks, while the other three benchmark MO_x_ exhibited negligible basic property. For the CO_2_ desorption on CeO_2_, two major CO_2_ desorption peaks appear at 110 and 234°C, representing the weak and medium basic sites, respectively [32,33]. Furthermore, the CeO_2_ material exhibits weak high-temperature CO_2_ desorption signal (400–600°C), which might be attributed to the strong interaction of CO_2_ and CeO_2_ [33]. The basicity of the catalyst is believed to benefit the adsorption and catalytic activation of CO_2_ in CO_2_ reduction process. The porosity of MO_x_ is highly related to the diffusion of reactants and exposure of active sites. As summarized in table 1, the CeO_2_ exhibited the most abundant pores, which might contribute to the CO_2_ diffusion, chemisorption and then activation in ICCU.

**Table 1.** Pore information of M O_x_ materials.

materials	BET surface area m^2^ g^−1^	pore volume cm^3^ g^−1^
ZrO_2_	5.46	0.02
TiO_2_	11.85	0.03
CeO_2_	65.76	0.25
Al_2_O_3_	0.65	<0.01

### 3.2. Integrated CO_2_ capture and reverse water–gas shift reaction performance over MO_x_-CaO dual functional materials

**Figure 3.**
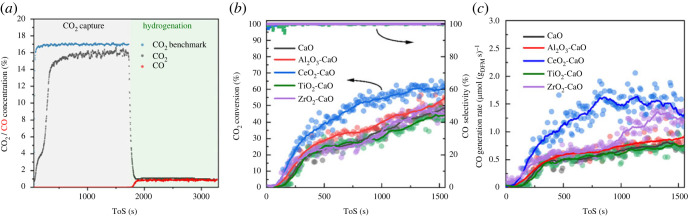
ICCU-RWGS performance of CeO_2_-CaO at 650°C (*a*); real-time CO_2_ conversion and CO selectivity (*b*) and CO generation rate (*c*) of ICCU-RWGS over MO_x_-CaO (M = Al, Ce, Ti or Zr) DFMs at 650°C.

The real-time catalytic performances of ICCU-RWGS using MO_x_-CaO (M = Al, Ce, Ti or Zr) DFMs are presented in figure 3. The Al_2_O_3_ TiO_2_ and ZrO_2_ are widely recognized as inert materials in thermal catalytic processes, which are applied as the benchmark in this work. To strictly exclude the potential effects of inert metal oxides on ICCU-RWGS, 0.2 g CaO without any MO_x_ (M = Al, Ce, Ti or Zr) was also evaluated for comparison. In the previous work [19,26], 650°C was suggested as the optimal temperature for CaO in ICCU based on the carbonation-decarbonation kinetics. Herein, the ICCU-RWGS evaluations using physically mixed MO_x_ and CaO formed DFMs (MO_x_-CaO) were firstly carried out at 650°C (figure 3).

As shown in figure 3*a*, the CaO in DFMs capture CO_2_ via carbonation from a 100 ml min^–1^ 17% CO_2_/N_2_ gas mixture (simulating flue gas) for 1700 s. After that, the carbonated MO_x_-CaO DFMs were reduced in a 5% H_2_/N_2_ at 100 ml min^–1^ for reverse water–gas shift reaction (RWGS), as shown in figure 3*a*. The real-time carbonated CO_2_ conversion possessed a gradually increasing trend as a function of time in the hydrogenation step. In the initial stage, the CO_2_ conversion was hindered by the fast CO_2_ release rate ascribed to the rapid decomposition of surface carbonates [19], which decreases the H_2_ partial pressure (i.e. concentration) around DFMs and hinders the CO_2_ conversion with H_2_. With the consumption of the surface carbonates, the subsurface carbonate species exhibit a slower CO_2_ release rate, then the increased H_2_ partial pressure over the surface of the DFMs enhanced CO_2_ hydrogenation performance with increased CO_2_ conversion as shown in figure 3*b*. The inhibition of CO_2_ release on the enhancement of the catalytic performance can be further evidenced by the real-time CO generation rate (figure 3*b*). In the initial approximately 250 s hydrogenation, the CO generation rate was even lower than 0.5 μmol g_DFM_ s^−1^ on all tested DFMs. Notably, all the tested DFMs exhibited excellent CO selectivity (greater than 99%), which outperformed the commonly applied Ni-based DFMs [19].

The Al_2_O_3_-CaO and ZrO_2_-CaO DFMs, assigned as inert benchmarks, possessed similar ICCU-RWGS performance compared with CaO-alone. In the previous research [26], the CaO-alone was proven active for ICCU-RWGS via direct hydrogenation of carbonates. It can also be concluded that TiO_2_ is inactive in ICCU-RWGS according to the poor performance of TiO_2_-CaO DFM. And the interaction between TiO_2_ and CaO (CaTiO_3_) performs no promotion effect on CO_2_ hydrogenation in ICCU. The CeO_2_-CaO outperformed all the other tested DFMs and possessed superior catalytic CO_2_ conversion (approx. 60%) and CO generation rate (approx. 1.5 μmol g_DFM_ s^−1^) at 650°C (figure 3*c*).

### 3.3 The effect of CeO_2_ mass fraction on integrated CO_2_ capture and reverse water–gas shift reaction performance

**Figure 4.**
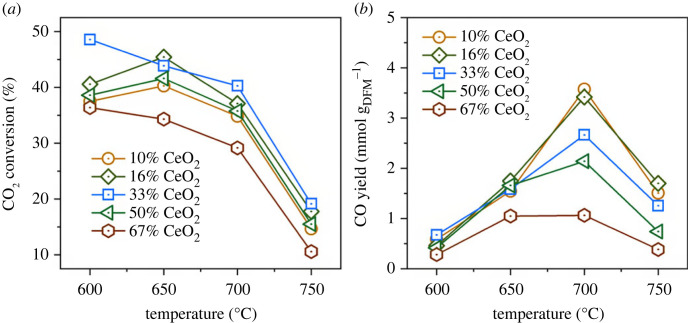
The effect of CeO_2_ fraction in CeO_2_-CaO DFMs over ICCU-RWGS at various temperature: (*a*) CO_2_ conversion and (*b*) CO yield.

To identify the optimal fraction of CeO_2_ in the CeO_2_-CaO DFM, a set of DFMs (CeO_2_ fraction: 10–67 wt%) were evaluated for ICCU-RWGS performance at a temperature range of 600°C to 750°C (figure 4). The 33 wt% CeO_2_ in DFM exhibited the optimal CO_2_ conversion at the tested temperatures. The low CeO_2_ fraction can restrict the catalytic performance due to the insufficient catalytic sites, while a CeO_2_ fraction over 50% can lead to a restriction of the adsorbent amount and hinder the overall CO_2_ capture performance of DFM. As can be seen from the CO yield results in figure 4*b*, a lower CeO_2_ fraction, representing a higher CaO fraction, could provide more carbonates and thus achieve a higher CO yield. For example, 10% and 67% CeO_2_-CaO DFMs achieved 3.5 and 1.0 mmol g_DFM_^−1^ CO yield at 700°C, respectively.

The reaction temperature is another key parameter in ICCU-RWGS using CeO_2_-CaO DFMs. The RWGS is an endothermic reaction [34], which prefers a higher temperature. However, the simultaneous decomposition of CaCO_3_ is more intense to release excessive CO_2_ at higher reaction temperature [35], especially in the tested temperature range. The trade-off between RWGS performance and carbonates decomposition can reflect on CO_2_ conversion and CO yield (figure 4). As shown in figure 4*a*, the CeO_2_-CaO DFMs possessed relatively higher CO_2_ conversion and reasonable CO yield at 650°C in approximately 1700 s hydrogenation, which is proven a suitable temperature for ICCU-RWGS.

### 3.4. Cycle performance of integrated CO_2_ capture and reverse water–gas shift reaction using 33% CeO_2_-CaO dual functional material

**Figure 5.**
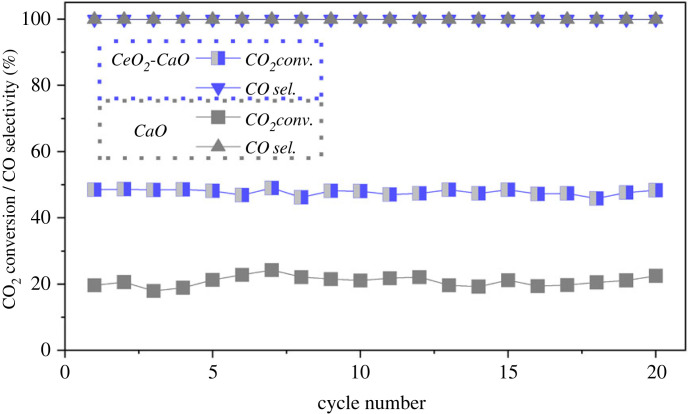
Cycle performance of ICCU-RWGS using 33% CeO_2_-CaO DFM at 650°C.

**Figure 6.**
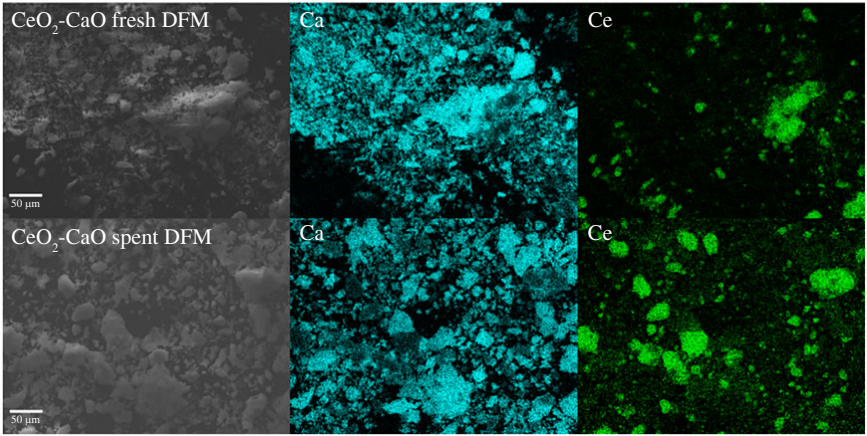
SEM-mapping images of fresh and spent (after 20 cycles) CeO_2_-CaO DFM.

The cycle stability of the optimal 33% CeO_2_-CaO DFM was further evaluated on ICCU-RWGS at the determined suitable reaction temperature of 650°C. As shown in figure 5, 33% CeO_2_-CaO possessed impressive stable catalytic performance in 20 reaction cycles. Specifically, the CO_2_ conversion and CO selectivity using 33% CeO_2_-CaO were sustained at approximately 49% and greater than 99%, respectively. As shown in figure 6, the morphology and chemical composition of CeO_2_ and CaO in the catalyst were stable after cyclic evaluation, indicating the outstanding stability of CeO_2_-CaO DFM in ICCU-RWGS. Furthermore, the 33% CeO_2_-CaO DFM possessed superior catalytic activity (approx. 49%) compared with CaO-alone, which achieved only approximately 20% CO_2_ conversion during ICCU-RWGS cycles.

In the previous research, the CeO_2_ possessed excellent synergistic catalytic performance due to the oxygen vacancy and abundant coordination defect [18,23,36]. In ICCU-RWGS, the CeO_2_ can provide a basic surface (figure 2*c*) to promote CO_2_ chemisorption and activation [23]. It is speculated that the CO_2_ is decomposed from carbonates, adsorbed on the surface of CeO_2_ and activated by its oxygen vacancies [37]. It is also believed that H_2_ could interact with ceria to promote CO_2_ hydrogenation [38,39]. Notably, the CeO_2_-CaO DFM can effectively and selectively (greater than 99%) convert CO_2_ into CO in the absence of active metals (e.g. Ni and Ru). It is known that CH_4_ formation is highly related to H_2_ dissociation [40,41], which hardly occurred in the absence of H_2_-sensitive active metals. Although some metals can achieve excellent CO selectivity in ICCU-RWGS via the redox pathway [19], such as Fe, a new drawback arises. Specifically, the Fe will be in the form of a metallic state after hydrogenation, which leads to undesirable CO formation in the following CO_2_ capture (equation (3.1)). Compared with that, the CeO_2_-CaO DFM demonstrated in this study is evidenced to address the above obstructions on undesirable CO formation but with superior and sustainable catalytic activity to realize a more promising ICCU-RWGS process.

3.1 $$M+XCO_{2}\to XCO+MO_{X}\;(M=\mathrm{Fe},\;\mathrm{Co}\;\mathrm{etc}.).$$

## 4. Conclusion

ICCU is an emerging process, which provides a more direct path between CO_2_-contained exhaust gas and catalytic conversion. The existing DFMs for ICCU-RWGS reaction meet obstructions on low CO selectivity, catalytic efficiency or undesirable CO generation in CO_2_ capture. Herein, a non-active metal containing CeO_2_-CaO DFM was synthesized to overcome the obstructions using simple and easy-access physically mixing method. The investigation of the effect of different MO_x_-CaO (M = Al, Ce, Ti or Zr) DFMs with different mass fraction of MO_x_ show that CeO_2_-CaO DFM with 33 wt% of CeO_2_ possessed significantly enhanced and sustainable (20 reaction cycles) ICCU-RWGS catalytic performance. Specifically, approximately 49% CO_2_ conversion and approximately 100% CO selectivity were achieved over the 33% CeO_2_-CaO DFM. As a comparison, CaO-alone could only realize approximately 20% CO_2_ conversion. The superior surface basicity on CeO_2_ is believed to contribute to CO_2_ chemisorption and activation among all tested MO_x_ (M = Al, Ce, Ti or Zr). The CeO_2_-CaO DFMs exhibit a cost-effective, noble metal-free, stable and highly efficient materials candidates for promising industrial application of ICCU.

## Data Availability

This article has no additional data.
